# Using the Person-based Approach to plan and develop an intervention for healthcare professionals to facilitate family-centred conversations when an adult patient has a serious illness

**DOI:** 10.1186/s12909-026-08767-x

**Published:** 2026-03-11

**Authors:** Elizabeth Rapa, Jeffrey R. Hanna, Sophie Ratcliffe, Rob Hallifax, Jack Arthey, Louise J. Dalton

**Affiliations:** 1 Department of Psychiatry, University of Oxford, Oxford, UK; 2 School of Nursing and Paramedic Science, Ulster University, 2-24 York Street, Belfast, UK; 3 South Eastern Health and Social Care Trust, Ulster Hospital, Dundonald, UK; 4 Faculty of English, University of Oxford, Oxford, UK; 5 Centre for Respiratory Medicine, Oxford University Hospitals NHS Foundation Trust, Oxford, UK; 6 University of Oxford, NIHR Oxford Biomedical Research Centre, Oxford, England, UK

**Keywords:** Children, Serious illness, Communication, Healthcare professionals, Family-centred conversations

## Abstract

**Background:**

Everyday thousands of adults across the world are diagnosed with serious health conditions. Many will have important relationships with children in their role as parents or grandparents. Communicating effectively with children about parental illness is associated with better psychological outcomes for children and family functioning. Adult patients report wanting help from healthcare professionals (HCPs) to think about sharing their diagnosis with children. HCPs describe feeling uncertain and unskilled in asking patients about their relationships with children; consequently support for patients is often absent.

**Aims:**

Plan and develop an intervention to enhance HCPs’ knowledge, skills and confidence about initiating family-centred conversations when an adult patient has a serious illness.

**Methods:**

The research used the Person-based Approach. Phase one: (1) Focus groups with adults with experience of their own or a partner’s illness and have a relationship with children (*n* = 12); (2) Focus groups with adults with lived-childhood experience of an adult’s illness (*n* = 6); (3) Individual interviews with professionals with a role in HCP training in a UK NHS setting (*n* = 9); (4) Focus groups with HCPs with experience of working in a UK NHS setting with adult patients who have an illness (*n* = 10). Phase two: ‘Think-aloud’ individual interviews with (1) adults with experience of their own or a partner’s illness (*n* = 2); (2) adults with lived-childhood experience (*n* = 2); (3) child with lived-experience (*n* = 1); (4) HCPs (*n* = 4). Focus groups and interviews were audio-recorded and transcribed.

**Results:**

Phase one: data were analysed using reflexive thematic analysis to identify the content for the intervention. Findings were triangulated and used to develop guiding principles and logic model to inform development of the draft intervention. The format selected was an animation. Phase two: Data were collected through 3 cycles with changes made to the audio, speed and visuals of the draft animation.

**Conclusions:**

The Person-based Approach facilitated the inclusion of multiple perspectives from different stakeholders. This extensive and rigorous process resulted in the development of an intervention in the form of an animation; evaluation is now required to assess the extent to which the animation increases HCPs’ knowledge, skills and confidence to initiate family-centred conversations with adult patients.

**Clinical trial number:**

Not applicable.

**Supplementary Information:**

The online version contains supplementary material available at 10.1186/s12909-026-08767-x.

## Background

Everyday healthcare professionals (HCPs) have to tell adult patients that they have a serious health condition. Serious illness can be defined as a condition that “carries a high risk of mortality and either negatively impacts a person’s daily function or quality of life or excessively strains their caregivers” [1]. It is estimated that 20 million people were diagnosed with cancer in 2022 [2] and chronic respiratory diseases affect over 500 million people worldwide [3]. Many patients who are told that they have a serious illness are parents, grandparents or have other significant relationships with children [4]. Telling children about the illness of a significant adult in their lives can pose a daunting challenge [5]. Families have a strong desire to protect children from the potentially devastating news and report uncertainty about what, how and when to talk to children [6, 7]. Consequently, adults may avoid telling children about a diagnosis [6]. However, even very young children (under the age of 5 years) are “astute observers” and notice subtle changes in adults’ behaviour or emotional state when someone becomes unwell [5, 8, 9]. Without a coherent explanation for their observations, children may draw their own (potentially inaccurate) conclusions and struggle alone [10]. Providing children with age-appropriate information enables them to understand what is happening and obtain emotional support [11, 12]. Global evidence indicates that effective communication is associated with lower reported child anxiety, depression and behavioural problems [5]. Furthermore, talking with children has benefits for parental mental health, treatment adherence and family functioning [5]. Inadequate information and poor communication between families and HCPs has been shown to increase the long-term risk for psychological distress (including risk of suicide attempts, self-injury and mortality) for those who were children during the adults’ illness or bereavement [13].

Research shows that adult patients want guidance from HCPs regarding how to talk to children about illness, but often feel unable to raise the topic with their healthcare team [14, 15]. Studies exploring HCPs’ perspectives outline a number of obstacles which frequently result in patients not receiving guidance or support about talking to their children [14, 16–19]. These include: (1) insufficient knowledge and confidence to initiate conversations with patients about their children (2) fears of upsetting the patient or children, or “making the situation worse” (3) uncertainty about whether this support constitutes part of their role (4) an assumption or hope this is addressed by a colleague (5) an absence of specific resources to discuss with patients about how, why and when children need to be told the diagnosis (6) a desire for training opportunities to enhance knowledge and feelings of competence (and thus confidence) [14]. Interventions are therefore required to address these barriers and facilitate HCPs to meet the needs of patients and their families [16, 20–22]. Research indicates that HCPs often erroneously assume colleagues elsewhere in the patients’ care pathway are addressing family-centred communication [16]. Interdisciplinary training may therefore be a step towards ensuring this important aspect of care is intentionally and routinely included within the healthcare system [21]. Collectively this evidence highlights a gap between patients’ needs and HCPs’ preparedness to support family-centred communication, indicating a need for brief, clinically-feasible training interventions.

The Person-based Approach provides a methodological framework for the development of health-related interventions that aim to promote behaviour change [23]. Extensive qualitative research is essential to identify and understand the context and beliefs held by the end-users so that these are reflected by the intervention [23]. The beliefs, needs and challenges identified through this preparatory planning work are then translated into guiding principles; these summarise the key objectives of the intervention and what features are required to achieve these.

In the context of family-centred care, the intervention must consider the perspectives of both those who will directly use it (i.e. HCPs) but also the ultimate recipients of the desired practice change initiated by the intervention (i.e. patients and children affected by an adult’s serious illness) [24]. Qualitative research throughout development ensures the resulting product is grounded in an appreciation of the users’ context and crucially their perspective of the behaviour change targeted by the intervention [24]. Developing an intervention with end-users has been demonstrated to achieve better retention rates to engagement, improved learning outcomes and efficacy of the user, with demonstrable positive impacts for clinical practice [23].

Using the Person-based Approach [25], this research aimed to plan and develop an intervention to enable HCPs to be equipped with the core principles, skills and confidence to facilitate family-centred conversations when a patient with significant relationships with children (< 18 years old) has a serious health condition.

### Objectives

1. Work with key stakeholders (families with lived-experience of serious illness and HCPs) to identify the fundamental attributes (content, structure, format) in planning an intervention for HCPs.
2. Identify challenges that must be addressed in the planning of an intervention to ensure successful use, dissemination and implementation.
3. Iterative development of an acceptable and implementable intervention.

## Methods

### Context

A Patient and Public Involvement (PPI) group was convened at study inception (May 2020) comprising five individuals (4 female, 1 male) with lived-experience of their own, or a partner’s serious illness including leukaemia, cancer and inflammatory disease. One member also had lived-childhood experience of parental serious illness. The PPI group were parents to children ranging from 3 to 16 years at the time of the study. One of the group was subsequently invited to join the project team as a PPI co-investigator and is a named co-author (JA). JA has lived-experience of a serious illness with a significant relationship with their grandchild, who was aged 18 at the time of the diagnosis. The PPI group members provided feedback on the grant application (including study design), commented on drafts of the participant information sheets to enhance readability, reviewed the topic guides for the focus groups (suggesting ways to make these more engaging for participants) and contributed to the planning and development stages.

### Study design

Underpinned by the Person-based Approach [23] methodology, the study had two phases:

Phase one: Using qualitative design methodology, interviews and focus groups with patients, partners, adults with lived-childhood experience and HCPs were conducted to elucidate the purpose, content and format of the intervention. Findings were used to develop guiding principles and logic model for the intervention. The intervention was subsequently designed in accordance with these frameworks.

Phase two: Individual ‘think-aloud’ interviews were conducted with HCPs, patients, partners and adults with lived-childhood experience to refine the intervention (developed in Phase one).

### Setting

Participants were identified from relevant professional and charitable organisations across the UK. To promote accrual, an advert was placed on the research Team’s professional accounts and social media platforms such as ‘X’. Furthermore, the team actively approached local faith leaders, the Filipino Nurses Association UK and Black, Asian and Minority Ethnic (BAME) networks within NHS Trusts and charities primarily (but not exclusively) supporting patients from different ethnic groups (e.g. Can-Survive UK).

### Population

Four population groups were included in Phases one and two comprising of 46 participants:

1. Adults with experience of their own or a partner’s serious illness (either ongoing or in the past) who have a relationship (e.g. parent, grandparent) with children, under the age of 18 years during the illness.
2. Adults with lived-childhood experience of an adult’s (e.g. parent’s, grandparent’s) serious illness during their own childhood.
3. Professionals with a role in the training of HCPs working with adult patients who have a serious illness in a UK NHS setting.
4. HCPs with experience of working with adult patients who have a serious illness in a UK NHS setting.

Phase two also included children under the age of 18 years with lived-experience of an adult’s (e.g. parent’s, grandparent’s) serious illness.

### Sampling

Phase one and two used purposive sampling to ensure a range of patients, partners, adults with lived-childhood experience of illness (including cancer, cardiomyopathy and stroke) and HCPs were involved (Tables 1 and 2). Professionals with a specialist role in HCP training and HCPs working in generalist and specialist roles across community and acute settings were also recruited using purposive sampling (Tables 1 and 2). Volunteer sampling techniques were also used to recruit patients, partners and adults with lived-childhood experience. Additionally, Phase two used purposive sampling to recruit children with lived-childhood experience through their ill parent (Table 2). Stakeholder engagement was led by members of the research team with experience of working in the NHS and by the PPI group and PPI Co-Investigator.

**Table 1:** Outline characteristics of the 37 participants recruited to phase one

Inclusion criteria	Patients and partners with lived-experience of a serious illness (*n* = 12)	Adults with lived-childhood experience (*n* = 6)	Professionals with role in HCP training (*n* = 9)	HCPs (*n* = 10)
	Experience of their own or a partner’s serious illness (either ongoing or in the past) Dependent child(ren) (under the age of 18 during illness) Sufficient verbal English to participate in a focus group	Experience of a parent’s serious illness during their own childhood Sufficient verbal English to participate in a focus group	Role in the core or ongoing professional training of HCPs	Experience of working with adult patients (age 18 years and older) who have a serious illness in a UK NHS setting
Participants	Current patient (*n* = 7) Patient and partner of patient (*n* = 2) Partner of patient (*n* = 3) *Role at time of illness*: Parent (*n* = 4) Grandparent (*n* = 4) Parent to children < 18 yrs & Grandparent (*n* = 3) Godparent (*n* = 1)	Daughter of patient (*n* = 5) Granddaughter of patient (*n* = 1)	Director of a UK Centre for Education and Research in Palliative Care Training Programme (*n* = 1) Lead for UK cancer charity (*n* = 1) Head of a National School of Healthcare Science (*n* = 1) Academic Training Programme Director of UK University Medical School (*n* = 1) Director of Learning at a Royal College (*n* = 1) Education Lead of a UK Centre for Education and Research in Palliative Care (*n* = 1) Head of Learning Innovation at a Royal College (*n* = 1) Course Lead in School of Nursing and Midwifery in UK University (*n* = 1) Divisional lead for Practice Development and Education in an NHS trust (*n* = 1)	Advanced Critical Care Practitioner (*n* = 1) Consultant Endocrinologist (*n* = 1) Foundation Year Three Doctor (*n* = 1) Genetic Counsellor (*n* = 1) Oncology Clinical Nurse Specialist (*n* = 4) Physiotherapist in Critical Care (*n* = 1) Registrar in prehospital Emergency and Critical Care/A&E trainee (*n* = 1)
Age of children at time of adult’s diagnosis	0–11 yrs (*n* = 19) 12–18 yrs (*n* = 10)	0–11 yrs (*n* = 3) 12–18 yrs (*n* = 3)	X	X

**Table 2:** Outline characteristics of the nine participants recruited to phase two

Inclusion criteria	Patients and partners with lived- experience of a serious illness (*n* = 2)	Adults with lived-childhood experience (*n* = 2)	Children with lived-childhood experience (*n* = 1)	HCPs (*n* = 4)
	Experience of their own or a partner’s serious illness (either ongoing or in the past) Dependent child(ren) (under the age of 18 during illness) Sufficient verbal English to participate in an interview	Experience of a parent’s serious illness during their own childhood Sufficient verbal English to participate in an interview	Experience of a parent’s serious illness during their own childhood Sufficient verbal English to participate in an interview	Experience of working with adult patients (age 18 years and older) who have a serious illness in a UK NHS setting
Participants	Current patient (*n* = 2) Role at time of illness: Parent (*n* = 2)	Daughter of patient (*n* = 2)	Daughter of patient (*n* = 1)	Clinical Nurse Specialist (*n* = 1) ICU nurse (*n* = 1) GP (*n* = 1) Consultant in Palliative Care (*n* = 1)
Age of children at time of adult’s diagnosis	0–11 yrs (*n* = 1) 12–18 yrs (*n* = 3)	12–18 yrs (*n* = 2)	12–18 yrs (*n* = 1)	X

### Recruitment

Individuals expressed interest by responding to the recruitment advert and emailing the senior author (LJD) who provided them with the participant information sheet outlining the purpose of the study and what participation would involve. Interested and willing individuals were recruited to the studies by return of a consent form and arranging attendance at an online focus group or individual interview (Phase one) or individual ‘think-aloud’ interview (Phase two).

Recruitment of professionals with a specialist role in HCP training additionally included targeted recruitment using the research team’s existing network, and snowball recruitment through participants’ own networks.

### Data collection

#### Phase one

##### Patients, partners and adults with lived-childhood experience

A total of seven patients, five partners and six adults with lived-childhood experience were recruited to one of three focus groups conducted in July and August 2022. A topic guide (appendix 1) was developed by the research team, informed by the literature and the PPI group. The two focus groups for patients and partners were facilitated by ER and LJD (duration 2 h 42 min) and LJD and JA (duration 2 h 37 min). The focus group for adults with lived-childhood experience was jointly facilitated by ER and SR with LJD acting as an observer and notetaker (duration 2 h 36 min). Focus groups were all audio-recorded.

##### HCPs

Five professionals with a role in HCP training responded to the advert and were recruited. These individuals identified an additional four people from their professional network who were contacted by ER about participating. Thus nine professionals working in relevant healthcare education roles across the UK participated in individual interviews, conducted in person (*n* = 3) and online (*n* = 6) by ER and LJD between July and September 2022. The interviews lasted between 30 and 46 min (mean = 39 min). The interviews explored participants’ views about the key attributes of a training intervention for their professional group and their perception of previously successful (or unsuccessful) training initiatives. Perspectives about the design features of an intervention to promote dissemination and adoption were also specifically explored.

A total of ten generalist and specialist HCPs from a range of clinical disciplines were recruited and participated in one of two online focus groups. Prior to the focus group participants were given background information about the study, including high-level detail about the challenges reported by families. The topic guide (appendix 2) was developed by the research team, informed by the literature and the PPI group. Participants were encouraged to share their perception and experiences of family-centred communication, the associated challenges and what HCPs needed to address these. The two focus groups were conducted in September 2022 (lasting 59 and 58 min respectively) and were jointly facilitated by ER and LJD, with RH acting as an observer and notetaker. Focus groups were audio recorded.

#### Phase two

A total of nine patients, partners, adults and children with lived-childhood experience and HCPs participated in individual, one-off ‘think-aloud’ interviews. These were conducted online in January 2023 by ER and LJD and lasted between 21 and 32 min (mean = 22 min). Three participants individually “walked through” the draft of the intervention (draft animation and draft audio). They were prompted to talk through their perception and understanding of each scene and encouraged to provide critical feedback and suggestions for improvements or clarifications. These were noted down by the interviewer. The intervention storyboard illustrations, text and audio were refined and presented to a second set of new participants (*n* = 4) for another cycle of individual interviews as described above. Minor changes were made in response to their feedback. The next version was shown to two further new participants who did not identify any suggestions or changes.

### Data analysis

#### Phase one – interviews and focus groups

Audio-recordings were transcribed verbatim using automated live audio transcription and accuracy-checked by the research team (ER and LJD). Reflexive thematic analysis guided the analysis [26, 27]. Initially, ER and LJD read and re-read the transcripts to gain a sense of each participant’s contributions and experiences. Deployed as an inductive method, codes were developed by marking similar phrases or words in the narratives. ER and LJD undertook an analytical mind mapping process to explore patterns and relationships in the data and to identify themes [28, 29]. These outputs were separately analysed by ER and LJD and refined through critical dialogue with JA, SR and RH. To promote study trustworthiness, the findings were shared with the participants by email. Five participants responded to confirm that the findings reflected their experiences, and no participants responded with amendments or disagreement about the summaries.

The focus group data were then triangulated across the three participant groups (patients and partners with serious illness, adults with lived-childhood experience, HCPs) to develop a broader, deeper understanding of the content and presentation of the intervention. Investigator triangulation [30] as deployed by involving multiple researchers (initially ER, LJD and JRH, followed by critical dialogue and discussion with SR, JA and RH) in the data analysis ensured different viewpoints and perspectives were represented and included throughout the process.

A summary of the recommendations and outputs from Phase one were collated and requirements for the intervention discussed with three animators and film makers with previous experience of producing NHS resources. shCreative aligned most closely with the intervention requirements and was commissioned to produce the intervention.

#### Phase two – qualitative ‘think-aloud’ interviews.

Interviewer notes detailing participants’ suggestions for changes were collated into an excel spreadsheet and discussed with the study team (LJD, ER, JA, RH, SR). Amendments identified in each cycle were then shared with the animator who enacted the changes. No further changes were identified in cycle three of user-testing of the intervention.

### Ethical considerations

Participants were presented with oral and written information about the study and provided written, informed consent. Participants were aware of the right to withdraw at any stage, with data protection procedures being observed, and given assurances of confidentiality. Recruiting adults with lived-childhood experience in Phase one enabled inclusion of both participants who had, and had not, been informed about their significant adult’s illness at the time; including this range of communication experiences enhanced the richness of the focus group discussion and facilitated consideration of all perspectives. In Phase two children under 18 years old with lived-experience of a parent’s illness were invited to participate; they could choose if they wanted a parent present during the interview. Participants’ wellbeing remained a priority throughout and this was monitored during the focus groups which all included an experienced Clinical Psychologist (LJD). Individual follow-up to address any distress arising from the research activity was offered to all participants but was not required. PPI group members and research participants were reimbursed for their time spent contributing to the studies in line with NIHR recommendations. Ethical approvals were obtained from University of Oxford Central University Research Ethics Committee (R74959).

## Results

### Phase one – Interviews with professionals working in relevant healthcare education roles

Individual interviews with professionals explored the key characteristics of a training resource that would be acceptable and accessible to HCPs. Participants acknowledged a wide spectrum of professional practice around family-centred conversations. This ranged from HCPs’ beliefs that this might be a valuable aspect of their (or another colleague’s) role, to those who had fully integrated this aspect of care. The participants identified the challenge of developing an inclusive intervention that could engage and energise HCPs holding different beliefs about the importance of family-centred conversations. To address perceived obstacles some participants proposed that the intervention should focus on the evidence regarding the benefits of family-centred communication for patients and families. Others believed it would be more effective to use real stories to help HCPs connect at an emotional level with the importance of this work.

*“I think stories can communicate. They can cut through even the most resistant individuals. Everyone can then lock onto the story”* (Professional with role in HCP training Participant ID 108).

Presenting the content from the perspective of the HCP and explicitly acknowledging the often-cited obstacle of time were also suggested as ways to ensure the intervention was relatable and resonated with HCPs. Many participants highlighted the multiple demands on their colleagues and emphasised that the intervention must be easy to access and succinct.

*“People’s time has*,* time and time again*,* come back as something that has been sparse. So chunks of material rather than looking at*,* 7 hours of e-learning…. How do we make it accessible for different people and to keep that vibrant and engaging*,* and at the same time so people can do it on a bus or in a 20-minute lunch break”* (HCP with role in professional training Participant ID 107).

Several participants suggested prioritising the rationale for family-centred conversations. They believed that once HCPs became aware of a gap in their own practice, they would then be motivated to independently seek additional (more comprehensive) training. Consequently, many recommended signposting links to further training opportunities. Providing written resources to accompany the intervention was also suggested to help HCPs feel more confident to embed family-centred conversations in their practice.

*“Once you worry HCPs they’ll go and find out the skills”* (Professional with role in HCP training Participant ID 101).

### Phase one – focus groups with Patients, Partners, adults with lived-childhood experience of an adult’s serious illness and HCPs.

#### Theme 1: challenges to family-centred conversations

Patients and their partners consistently reported that HCPs had focused on the physical aspects of the illness and had not enquired about whether they had any relationships with children. Consequently, these important family connections were overlooked, meaning that patients and their partners had not been offered any guidance or support about explaining their diagnosis and illness to children. This was consistent with the reports of adults with lived-childhood experience, who frequently described experiences of HCPs visiting their ill parent at home but not talking to them directly, or “*being ushered out of the room”* during a parent’s medical appointment. All the participants with lived-childhood experience had been aware of the adult’s illness, but in the absence of any explanation from family members or other adults described feeling fearful and confused. Consequently, many had obtained information by deliberately or accidentally overhearing adult conversations. Participants reflected that this had resulted in misunderstandings about the adult’s illness, or making a devastating discovery about a parent’s prognosis without emotional support to make sense of the news. Participants with lived-childhood experience described feeling excluded from the *“journey”* of their parent’s illness. The impact of serious illness both on the patient **and** their extended family was consistent with patients’ own perspective, with all expressing a desire for HCPs to acknowledge them (the patient) as part of a wider family network.

*“Children are in the house*,* they’re completely impacted by everything that’s happening*,* actually it should be the whole unit that goes on that journey and is experiencing it together. And for the consultant and staff to maybe just think about the fact that*,* we’re not an Individual*,* we are a ‘we’.”* (Adult with lived-childhood experience Participant ID 302).

The focus of clinical staff on “*the patient in front of them”* was repeatedly recognised by HCPs themselves, with most acknowledging that considering the impact of a patients’ illness on children was not part of their usual practice. HCPs described the challenge of juggling multiple, competing priorities in their limited time with patients. Professionals reported that this pressure meant that there was only sufficient time to address medical issues, rather than considering family-centred conversations. Alongside this, HCPs reflected that the emotional impact of these sensitive conversations on professionals could also contribute to an avoidance of this aspect of care. In addition, all HCPs expressed an implicit assumption that family-centred conversations with patients would be lengthy, reinforcing the perception of their incompatibility with current workload pressures.

“*You’re always rushing and there’s not enough time…. on a medical ward people will struggle to even start this conversation because they know they have*,* like*,* 20 more patients to see in the ward round and they cannot have that 20 minute conversation.*” (HCP Participant ID 401).

Several HCPs expressed a sense of uncertainty about whether family-centred conversations would be welcomed by their patients. HCPs reported that this stemmed from experiences of working with families who had explicitly decided not to tell children about an illness or diagnosis. Other HCPs reflected upon experiences where patients had *not* asked them about talking to children; from this they had assumed it was *not* an area of need for patients. Some HCPs expressed concern that patients could often feel “*overwhelmed”* with the volume of information that was routinely shared in appointments (particularly at the time of diagnosis) and therefore did not raise the topic of patients’ children.

*“You don’t want to feel like you’re imposing on someone’s life more than you already are when you’re already giving them a life-changing diagnosis.”* (HCP Participant ID 405).

Some HCPs reflected that their training was focused on adult patients and therefore were unsure about whether children should be told about an adult’s illness, or if it would be unnecessarily upsetting for children. This dilemma was echoed by some patients and partners who expressed their uncertainty about what was “*the right thing to do.”* In contrast, adults with lived-childhood experience described their loneliness and anger at not being told and “*kept out of the loop”* at the time of the adult’s illness. They described the ongoing legacy enduring for many years; this included sadness about missed opportunities to talk or spend time with a parent before their death.

“*Really do we tell them the truth? Do you try and hide it? He < grandfather> wasn’t playing because he wasn’t well. So it was really about how honest are you? Because you know we wanted to be realistic*,* but we didn’t want to frighten them*,* the girls*,* and it really is difficult to know*” (Partner of patient Participant ID 202).

Patients and partners reported not knowing how to talk to their children and wanted guidance about what to say and when these conversations should take place. Many HCPs also did not feel they had the skills to advise their patients on how to talk to children about an illness.

“*I’m an adult nurse*,* so I*,* I look after adults*,* so it’s really hard. you know*,* we don’t have the skills or the qualifications to deal with it.*” (HCP Participant ID 409).

#### Theme 2: needs that must be addressed to achieve family-centred conversations in routine care

Patients and partners highlighted the importance of asking patients of all ages about their relationships with children, with many emphasising the significance of their grandchildren. Participants with lived-childhood experience believed that information should be tailored to children’s age. In addition, these participants wanted parents to acknowledge and provide an opportunity to talk about the emotional implications of the information that was shared.

Parents and partners wanted clarity and reassurance from their medical team about whether to talk to children about the illness. They described feeling unsure about the quality and trustworthiness of information available from different sources, such as the internet. Patients and partners described wanting practical suggestions about how to talk with children. Similarly, HCPs highlighted their need for (1) an evidence-based rationale as to why families should tell children about the illness (2) practical tools to help initiate conversations in clinical practice or answer questions and concerns patients may have about talking to the children.

*“What you need sometimes as a HCP is to actually have the evidence to be able to confidently say to patients*,* “look*,* I know this feels like a very difficult conversation. Now I’m not the parent of your children*,* but this is what the evidence tells us and all I can say to you is it might not be ready now*,* but I think the best thing for everyone in this situation is to speak to your children”…. a few bite-sized headlines that can help to win people over to that…if you can say it with confidence…”* (HCP Participant ID 405).

HCPs suggested different approaches that could be helpful to remind clinicians of the importance of family-centred conversations. These included changes to clinical templates so that they included space to record patient relationships with children and what they had been told, and visual prompts with short, easy to remember steps or acronyms. Patients, partners and adults with lived-childhood experience also suggested similar initiatives both in terms of their clinical record, but also publicity posters in waiting rooms encouraging patients to think about what children needed to be told. HCPs felt that patients could play an important role in raising the topic of children with their clinician, suggesting the use of special badges on their ID lanyards which had been used successfully in the past to encourage patients to actively check clinicians had washed their hands.

*“Thinking back to some of the campaigns that have hit home and stuck over the last few years …. We all get a badge now that says ‘My name is….’; its been sort of drilled into us now that we need to introduce exactly who we are. There was a thing [for patients] saying ‘Don’t be afraid to ask ‘have you [HCP] washed your hands?’ And the next thing could be ‘Have you asked about my family situation?’ and that should be your automatic.”* (HCP Participant ID 403).

Patients, partners and adults with lived-childhood experience all reflected on the importance of keeping children updated about the illness over time. Adults with lived-childhood experience emphasised their need to be told even when the news was “*not good”*, expressing the importance of honest communication. Patients and their partners wanted an ongoing dialogue with their clinical team about family-centred communication, indicating that over the course of their illness (which could be many years) it would be helpful for HCPs to “*check-in”* about their children’s understanding.

*“It’s not just a one off ‘Right you’ve had this diagnosis you need to speak to your kids’-done- but actually keep doing it as a health professional: ‘How are you? How are you talking to kids? How’s it going?’. keep that conversation going throughout the process*,* throughout the journey rather than just ‘This is it*,* ticked the box*,* we’ve talked to them[{ patients] about telling the children’.*” (Patient Participant ID 202).

### Intervention development

**Fig. 1:**
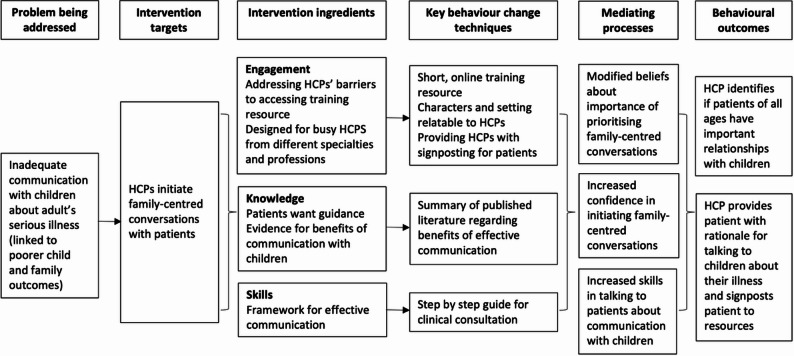
Logic model

The findings from Phase one were reviewed by the research team and used to inform guiding principles highlighting the key attributes and content for a successful intervention (Table 3). A logic model [23] was developed to illustrate the intended mechanism of change (Fig. 1).

**Table 3:** Guiding principles for the intervention

Intervention design objectives	Key features
To help HCPs recognise the importance of considering all patients’ family relationships	- Short intervention to maximise engagement from HCPs. - Highlight that patients do want to be asked by HCPs about family relationships. - Address HCP fears about time pressures. - Reassure HCPs and parents that children want to be informed about serious illnesses. - Inform HCPs of the key role adults (e.g. grandparents) play in children’s lives.
To enhance HCPs’ understanding of the impact of communication about serious illness on child and family outcomes	-Provide key evidence about the impact of effective communication on child and family outcomes. - Highlight the emotional impact on children when they are not explicitly told about the illness.
To provide HCPs with the skills to talk to patients about telling children about the illness	- Clear guidance and suggestions for HCPs about what, when and how to support patients to talk with their children. - Provide HCPs with additional evidence-based resources to enhance their learning. - Provide HCPs with additional resources so they can share these with families for further support and guidance.
To help HCPs from different specialties and backgrounds recognise they can play a role in contributing to family-centred care	-Provide content from perspective of HCP. -Encourage self-recognition by HCP of their role and working context.

The Phase one results, guiding principles and logic model determined that the intervention should be online so that it was readily accessible and easy to share. A text-based intervention was discounted due to the importance of quickly engaging busy HCPs who also might not consider the content to be of relevance to their role. The requirements for the intervention were then discussed with a number of filmmakers and animators. An animation was selected to facilitate inclusion of a large volume of information with key steps that could be easily highlighted within the narrative. This format enabled the content to reflect the key recommendations of the professionals with a role in HCP training; the inclusion of a ‘patient story’ to emotionally engage the viewer throughout, presented from the perspective of an HCP and interwoven with key evidence and practical recommendations. Animation maximised the opportunity for including a diverse range of characters (representing different genders, ages, regional accents, religious and ethnic groups) whilst minimising potentially distracting and irrelevant contextual cues (e.g. background details of a location) and maintaining an overall coherence to the story. A hand drawn animator was chosen as it felt this artistic style was consistent with the serious and sensitive nature of the subject matter and intended professional audience. This method also offered flexibility to change aspects of the visual appearance in response to the research team and participants’ feedback in Phase two.

The research team and animator (shCreative) worked in collaboration to iteratively develop and refine the script, visual images and audio content. This involved multiple meetings over a 6 week period to achieve a draft animation that could be shared with participants in Phase two of the study. Table 4 outlines the mapping of the Phase one results, intervention content and format to the logic model.

**Table 4:** Logic model behavior change targets, phase one results and intervention content and format

Intervention target and ingredients	Key behavior change target	Results from Phase one	Inclusion in the intervention
HCPs initiate family-centred conversations with patients	HCPs recognize family-centred communication is a patient priority for consultation	Priority for patients and their family HCPs are uncertain about whether patients want or need guidance HCP focus on patient in front of them and invisibility of wider family relationships Time pressure on HCPs	Patient quotes (voiced) highlighting the importance of their children in context of new diagnosis and desire for guidance from healthcare team about talking to children Visuals depict patients of wide age-range and with different responsibilities (i.e. parent, grandparent) Family around patient visually represented immediately after consultation with HCP Narrator acknowledges time pressure on HCPs
Engagement	Short, online training	Stories are engaging	5 min 2 s duration Design suitable for viewing on mobile phone Patient quotes (voiced) from perspective of a patient, partner and children Visuals represent HCPs in both their professional role and as a parent themselves
	Characters and setting relatable to HCPs		HCPs of different genders, age and ethnicity represented Narrator speaks from perspective of an HCP Generic healthcare backgrounds represented visually so viewer can project their workplace into visuals
	Provide HCPs with signposting for patients	Patients want practical support to use at home	Links to website for patients to download practical steps to talk directly to children
Knowledge	Summary of published literature regarding benefits of effective communication	HCPs and families unsure about risks and benefits of talking to children about illness	Audio and visual presentation of key literature review findings Partner’s quote (voiced) expressing dilemma of whether to tell children about adult’s illness Lived-experience quotes (voiced) highlighting negative impact of not being told about adult’s illness Scene depicts positive intergenerational impact of honest communication about adult’s illness
Skills	Step by step guide	HCPs will seek further training once aware of an issue Practical steps helpful for HCPs Importance of following up with families about communication with children during duration of illness	4 steps presented visually during animation and summarised at end Downloadable resource for HCPs to use Scene of HCP following up with patient about talking with children

### Phase two – qualitative ‘think-aloud’ interviews

Participants’ recommendations at each cycle of ‘think-aloud’ interviews and the subsequent changes to the storyboard and audio, are summarised in Table 5. Changes to both presentation and the content of the intervention were identified in cycles 1 and 2, with no further changes at cycle 3. No major changes were identified; participants’ recommendations focused on ways to clarify or enhance the existing messages.

**Table 5:** Changes and refinements made during phase two

	Participants’ recommendations cycle 1	Change enacted to intervention	Participants’ recommendations cycle 2	Change enacted to intervention
**Presentation** visuals and audio	Getting to Step 1 takes too long	Re-ordered first scenes to shorten time before first step presented to highlight practical implications of content presented	First patient looks too old	Wrinkles removed to make patient more relatable to HCPs working in different settings
	Gap between Step 1 and Step 2 too long	Transition speed increased	Hard to listen and read the results of the ‘Lancet scene’ at the same time	Audio matched with the words appearing
	Title of Step 2 too long	Removed ‘will find it helpful to have’		
	Skin tone of first HCP too pale	Changed HCP skin colour to enhance ethnic diversity of characters	Too quick from last patient voiceover to resources website	Inserted pause after ‘….not just me’ to emphasise verbal message
	Do not like all uppercase for Step titles	Changed to lowercase and just letter of first word capitalised to enhance readability	Some font is too pale	Re-worked font to be bolder and enhance readability
	Unsure if patient talking to HCP is the same one who then talks to his family	Clothing of first patient made more distinctive in colour to make story clearer		
**Content**	Needs to emphasise from the start that this intervention is relevant for HCPs from every discipline	Added ‘your’ before patient; Added ‘we as HCPs’ to voiceover; Added additional explicit references to HCPs throughout	Hard to remember all the steps	Show steps at the start and then repeated as a list near the end of the animation
			In Step 3 make it clear the HCP has to give the guide to their patient	Added the HCP giving the guide to patient in Step 3 image, and made sleeve of HCP more distinctive in subsequent scene
			Need clarification of why talking to patients about children is important to do	Added final still-frame repeating the evidence

The intervention is hosted and publicly accessible at https://youtu.be/0esY5bsLM84.

## Discussion

This paper documents the development of an intervention to equip HCPs with the knowledge, skills and confidence to initiate family-centred conversations using the Person-based Approach. Effective communication with children about an adult’s serious illness is associated with better psychological outcomes for both children and families [5]. Adults want guidance from their healthcare team [4, 31], but HCPs are uncertain whether these conversations would be welcomed, whether this is part of their role and feel they lack the skills to raise this topic with their patients [16]. Consequently, the topic of communication with children is often avoided in clinical appointments. The intervention therefore needed to engage HCPs and provide both the rationale for family-centred conversations and skills to undertake this aspect of care for HCPs. The interdisciplinary nature of the intervention is consistent with the recommendations of a recent systematic review for educational interventions to reflect the multidisciplinary care of patients and therefore enhance the quality of care [21].

The Person-based Approach [23] facilitated the inclusion of the voices of multiple stakeholders to ensure the content matched the potentially diverse informational needs of the target audience and included representatives from different professional groups. Their wide range of opinions emphasised the challenge of creating an intervention that would address the diversity of training needs and motivators for behaviour change across different individuals and professions. Some of the professionals with a role in HCP training reported that empirical evidence was of primary importance to *“convince”* HCPs of the need to prioritise family-centred conversations, while others believed that emphasising the emotional impact of family illness for children would be more significant. These results were instrumental in establishing the key features of the guiding principles for intervention development. It was important to balance the training needs of HCPs with the perspectives of patients, their partners and adults with lived-childhood experience. Involving adults with lived-childhood experience enabled recruitment of those who both had, and had not, been told about a significant adult’s illness during their childhood. This allowed an understanding of what had been helpful and also missed opportunities for communication. Identifying patients’ and partners’ dilemmas about telling children about the illness refined the key aspects which they wanted to be recognised and understood by HCPs.

A range of formats have been used for HCP communication skills training including eLearning manuals [24] and in-person training workshops [32, 33]. However, these often do not include specific material on having conversations with patients about communicating with children [32, 33] or exclusively focus on the advanced stages of cancer and end of life care (e.g. [24]). This leaves an important gap in resources for HCPs caring for the large number of patients with other serious health conditions for whom the issue of communicating with children remains extremely pertinent. The current study aimed to develop a generic intervention (i.e. not disease specific) that would encourage HCPs to initiate family-centred care from the point of diagnosis onwards (rather than at a much later stage in the illness trajectory).

Many illness-specific charities have written resources for parents about talking to children of different ages. Whilst leaflets and web-based booklets may be helpful, a qualitative study of mothers with breast cancer indicated that written materials were often unsuitable, out of date or overly negative [34], and a systematic review concluded that these cannot replicate the benefits of a personalised approach facilitated by a HCP [6]. Consistent with research [35], the PPI group and patients and partners reported that they felt overwhelmed with written information (related to their condition, treatment, financial support) from the medical team, particularly at diagnosis and consequently failed to read any or little of the material. The participants’ emphasised the importance of guidance from a trustworthy source such as their medical team, highlighting the pivotal role HCPs can play in facilitating families’ awareness and engagement about the importance of communication with children.

The results of the focus groups with HCPs in Phase one were consistent with previous research indicating the invisibility of patients’ relationships with children to clinicians [16]. Consequently, overstretched HCPs’ Continuing Professional Development priorities may not include enhancing skills and confidence around family-centred communication. The intervention design therefore needed to address the challenge of both addressing general barriers to training [36] but also reach and engage an audience who might not believe it would be of relevance or value to their role. Solutions to overcome generic barriers to upskilling HCPs include interventions that are short, easily accessible and memorable [36]. This is consistent with evidence supporting the effectiveness of Micro learning, which is used to describe short, discrete packages of educational content, often available online or in a format that can be easily stored and accessed on a smart phone [37]. The final intervention design was an animation which could be viewed ‘on the go’ or easily reviewed prior to a consultation. The digital, on-demand format offers the potential to be included as part of pre-existing training structures or events (e.g. statutory or mandatory training). Time constraints remain a barrier to engagement with digital educational tools [38]; the duration was minimised to facilitate its inclusion in training despite potentially skeptical audiences. It is hoped that the animation’s brevity could also facilitate leveraging the power of social media [39] for dissemination.

### Strengths and limitations

The strengths of this work include the engagement of leaders in HCP training and skills development and the range of participants’ professional background including physicians who are often poorly represented in research [40]. Participants in the study were recruited from across the UK, with the online format for focus groups facilitating wide geographical engagement. However, ethnicity data was not collected and the focus on UK-based HCPs working within the NHS may limit generalisability to other contexts and locations. Participants demonstrated their commitment to the study objectives through generously sharing their time and experiences. It is likely that those who participated had a pre-existing interest in the topic area or were open to exploring this area of practice; their views and experiences may differ to those who did not respond to adverts or invitations to participate.

## Conclusions

This paper describes the design and refinement of an intervention to facilitate family-centred conversations. Rigorous evaluation is now required to assess the extent to which the intervention achieves its identified goals of enhancing HCPs’ knowledge, skills and confidence to embed family-centred conversations as part of routine care. Future studies must then explore how these discussions within clinical settings influence families’ decisions and actions about talking with children when an adult has a serious illness.

## Supplementary Information

Supplementary Material 1

## Data Availability

Data are available on reasonable request from the corresponding author.

## Abbreviations

HCPs: Healthcare professionals
NHS: National Health Service
PPI: Patient and Public Involvement
UK: United Kingdom

## References

[1] Kelley AS, Bollens-Lund E. Identifying the population with serious illness: the “Denominator” challenge. J Palliat Med. 2018;21(S2):S7–16. 29125784 10.1089/jpm.2017.0548 PMC5756466

[2] 2. Bray F, Laversanne M, Sung H, Ferlay J, Siegel RL, Soerjomataram I, et al. Global cancer statistics 2022: GLOBOCAN estimates of incidence and mortality worldwide for 36 cancers in 185 countries. CA Cancer J Clin. 2024;74(3):229 − 63. 10.3322/caac.21834 38572751

[3] 3. Prevalence and attributable health burden of chronic respiratory diseases, 1990–2017: a systematic analysis for the Global Burden of Disease Study 2017. Lancet Respir Med. 2020;8(6):585 − 96. 10.1016/S2213-2600(20)30105-3 PMC7284317 32526187

[4] Rapa E, Hanna JR, Mayland CR, Mason S, Moltrecht B, Dalton LJ. Experiences of preparing children for a death of an important adult during the COVID-19 pandemic: a mixed methods study. BMJ Open. 2021;11(8):e053099. 10.1136/bmjopen-2021-053099 PMC8370837 34400462

[5] Dalton L, Rapa E, Ziebland S, Rochat T, Kelly B, Hanington L, et al. Communication with children and adolescents about the diagnosis of a life-threatening condition in their parent. Lancet. 2019;393(10176):1164–76. 30894272 10.1016/S0140-6736(18)33202-1

[6] Hanna JR, McCaughan E, Semple CJ. Challenges and support needs of parents and children when a parent is at end of life: a systematic review. Palliat Med. 2019;33(8):1017–44. 31244381 10.1177/0269216319857622

[7] 7. Tavares R, Brandao T, Matos PM. Mothers with breast cancer: A mixed-method systematic review on the impact on the parent-child relationship. Psychooncology. 2018;27(2):367 − 75. 10.1002/pon.4451 28477374

[8] 8. Beale EA, Sivesind D, Bruera E. Parents dying of cancer and their children. Palliat Support Care. 2004;2(4):387 − 93. 10.1017/s1478951504040519 16594401

[9] Forrest G, Plumb C, Ziebland S, Stein A. Breast cancer in the family–children’s perceptions of their mother’s cancer and its initial treatment: qualitative study. BMJ. 2006;332(7548):998–1003. 16613935 10.1136/bmj.38793.567801.AE PMC1450041

[10] 10. Stein A, Dalton L, Rapa E, Bluebond-Langner M, Hanington L, Stein KF, et al. Communication with children and adolescents about the diagnosis of their own life-threatening condition. Lancet. 2019;393(10176):1150-63. 10.1016/S0140-6736(18)33201-X 30894271

[11] Helseth S, Ulfsaet N. Having a parent with cancer: coping and quality of life of children during serious illness in the family. Cancer Nurs. 2003;26(5):355–62. 14710796 10.1097/00002820-200310000-00003

[12] Huizinga GA, van der Graaf WT, Visser A, Dijkstra JS, Hoekstra-Weebers JE. Psychosocial consequences for children of a parent with cancer: a pilot study. Cancer Nurs. 2003;26(3):195–202. 12832952 10.1097/00002820-200306000-00004

[13] 13. Li J, Vestergaard M, Cnattingius S, Gissler M, Bech BH, Obel C, et al. Mortality after parental death in childhood: a nationwide cohort study from three Nordic countries. PLoS Med. 2014;11(7):e1001679. 10.1371/journal.pmed.1001679 PMC4106717 25051501

[14] Hanna JR, McCaughan E, Beck ER, Semple CJ. Providing care to parents dying from cancer with dependent children: health and social care professionals’ experience. Psychooncology. 2021;30(3):331–9. 33091180 10.1002/pon.5581

[15] Semple CJ, McCaughan E, Smith R. How education on managing parental cancer can improve family communication. Cancer Nurs Pract. 2017;16(5):34–40.

[16] Dalton LJ, McNiven A, Hanna JR, Rapa E. Exploring healthcare professionals’ beliefs, experiences and opinions of family-centred conversations when a parent has a serious illness: a qualitative study. PLoS One. 2022;17(11):e0278124. 36441706 10.1371/journal.pone.0278124 PMC9704560

[17] Fearnley R, Boland JW. Communication and support from health-care professionals to families, with dependent children, following the diagnosis of parental life-limiting illness: a systematic review. Palliat Med. 2017;31(3):212–22. 27383635 10.1177/0269216316655736 PMC5347362

[18] Eklund R, Kreicbergs U, Alvariza A, Lövgren M. Children’s self-reports about illness-related information and family communication when a parent has a life-threatening illness. J Fam Nurs. 2020;26(2):102–10. 31931660 10.1177/1074840719898192

[19] Karidar H, Glasdam S. Inter-professional caring for children who are relatives of cancer patients in palliative care: perspectives of doctors and social workers. Br J Soc Work. 2018. 10.1093/bjsw/bcy080.

[20] Semple CJ, O’Neill C, Sheehan S, McCance T, Drury A, Hanna JR. An e-learning intervention for professionals to promote family-centered cancer care when a significant caregiver for children is at end of life: mixed methods evaluation study. J Med Internet Res. 2024;26:e65619. 39657171 10.2196/65619 PMC11668990

[21] Sheehan S, Hanna JR, Drury A, McCance T, Semple CJ, O’Neill C. A systematic review of educational interventions to equip health and social care professionals to promote end-of-life supportive care when a parent with dependent children is dying with cancer. Semin Oncol Nurs. 2023;39(5):151474. 37481410 10.1016/j.soncn.2023.151474

[22] Hanna JR, Semple CJ. Mixed-methods evaluation of a face-to-face educational intervention for health and social care professionals to deliver family-centred cancer supportive care when a parent with dependent children is at end of life. Psychooncology. 2024;33(7):e6374. 38977423 10.1002/pon.6374

[23] Yardley L, Morrison L, Bradbury K, Muller I. The person-based approach to intervention development: application to digital health-related behavior change interventions. J Med Internet Res. 2015;17(1):e30. 25639757 10.2196/jmir.4055 PMC4327440

[24] O’Neill C, Hanna JR, Sheehan S, McCance T, Drury A, Semple CJ. Adapting and testing an eLearning resource for professionals to support families when a significant caregiver for children is dying with cancer. BMC Palliat Care. 2024;23(1):268. 39574104 10.1186/s12904-024-01601-5 PMC11580503

[25] Yardley L, Ainsworth B, Arden-Close E, Muller I. The person-based approach to enhancing the acceptability and feasibility of interventions. Pilot Feasibility Stud. 2015;1:37. 27965815 10.1186/s40814-015-0033-z PMC5153673

[26] Braun V, Clarke V. Reflecting on reflexive thematic analysis. Qual Res Sport Exerc Health. 2019;11(4):589 − 97.

[27] Braun V, Clarke V. One size fits all? What counts as quality practice in (reflexive) thematic analysis? Qual Res Psychol. 2020;18(3):328 − 52.

[28] Braun V, Clarke V. Using thematic analysis in psychology. Qual Res Psychol. 2006;3(2):77–101.

[29] Braun V, Clarke V. Reflecting on reflexive thematic analysis. Qual Res Sport Exerc Health. 2019;11(4):589–97.

[30] Denzin NK. The research act; a theoretical introduction to sociological methods, vol. xiii, 368 p. Chicago: Aldine Pub. Co.; 1970.

[31] Semple CJ, McCance T. Experience of parents with head and neck cancer who are caring for young children. J Adv Nurs. 2010;66(6):1280–90. 20546362 10.1111/j.1365-2648.2010.05311.x

[32] North West Coast Learning Collaborative. Standards and Guidelines for the provision of Advanced & Key Level Communication Skills Training Programmes [Available from: https://www.england.nhs.uk/north-west/wp-content/uploads/sites/48/2021/10/2019-NWCLC-Standards-Portrait-for-ACST-and-KS-programmes.docx.pdf. ] Accessed on 15 December 2024.

[33] Turner M, Payne S, O’Brien T. Mandatory communication skills training for cancer and palliative care staff: does one size fit all? Eur J Oncol Nurs. 2011;15(5):398–403. 21163700 10.1016/j.ejon.2010.11.003

[34] Turner J, Clavarino A, Yates P, Hargraves M, Connors V, Hausmann S. Oncology nurses’ perceptions of their supportive care for parents with advanced cancer: challenges and educational needs. Psychooncology. 2007;16(2):149–57. 17061311 10.1002/pon.1106

[35] Semple CJ, McCaughan E, Beck ER, Hanna JR. “Living in parallel worlds” - bereaved parents’ experience of family life when a parent with dependent children is at end of life from cancer: a qualitative study. Palliat Med. 2021;35(5):933–42. 33765868 10.1177/02692163211001719 PMC8114437

[36] Gasteiger N, van der Veer SN, Wilson P, Dowding D. Upskilling health and care workers with augmented and virtual reality: protocol for a realist review to develop an evidence-informed programme theory. BMJ Open. 2021;11(7):e050033. 10.1136/bmjopen-2021-050033 PMC8258595 34226234

[37] Zarshenas L, Mehrabi M, karamdar L, Keshavarzi MH, keshtkaran Z. The effect of micro-learning on learning and self-efficacy of nursing students: an interventional study. BMC Med Educ. 2022;22(1):664. 36071456 10.1186/s12909-022-03726-8 PMC9450813

[38] Borek AJ, Campbell A, Dent E, Moore M, Butler CC, Holmes A, et al. Development of an intervention to support the implementation of evidence-based strategies for optimising antibiotic prescribing in general practice. Implement Sci Commun. 2021;2(1):104. 34526140 10.1186/s43058-021-00209-7 PMC8441243

[39] Katz M, Nandi N. Social media and medical education in the context of the COVID-19 pandemic: scoping review. JMIR Med Educ. 2021;7(2):e25892. 33755578 10.2196/25892 PMC8043144

[40] Frerichs W, Geertz W, Johannsen LM, Inhestern L, Bergelt C. Child- and family-specific communication skills trainings for healthcare professionals caring for families with parental cancer: a systematic review. PLoS One. 2022;17(11):e0277225. 36350839 10.1371/journal.pone.0277225 PMC9645618

