# Supplementary material for: Using the Person-based Approach to plan and develop an intervention for healthcare professionals to facilitate family-centred conversations when an adult patient has a serious illness
Source: BMC Med Educ. 2026 Mar 11;26:658. doi: 10.1186/s12909-026-08767-x (PMC13104269; doi:10.1186/s12909-026-08767-x)
Supplement: Supplementary file 1 — Supplementary Material 1 [file 12909_2026_8767_MOESM1_ESM.docx]

**Supplementary material**

Appendix 1: Topic Guides for Group 1 (Adults with lived-childhood experience of an adult’s serious illness) and Group 2 (Adults with lived-experience of their own or a partner’s serious illness)

| Housekeeping, Introductions and icebreaker | Adults with lived-childhood experience (Group 1) | Adults with lived-experience of their own or a partner’s serious illness (Group 2) |
| --- | --- | --- |
| Personal experiences of an adult’s serious illness | Memories of adult’s illness. Age you experienced adult’s illness.  Communication from adults about what was happening  What was helpful in understanding what was happening around you?  What made your experience more difficult? | Experience of whether HCPs asked about relationships with children in family network.  Positive moments of care around talking to children. Opportunities for improvement in facilitating family-centred conversations. |
| Perception of what HCPs need to know based on your experiences | What do HCPs need to know about the experience of being a child in a family affected by serious illness?  What training do HCPs need? | What do HCPs need to know about the experience of being a parent/grandparent/partner with a serious illness?  Who should initiate about having family-centred conversations?  What training do HCPs need? |
| Response to previously documented obstacles for HCPs initiating family-centred conversations | What do you think about HCPs’ concerns that these conversations might be intrusive or upset patients? | What do you think about HCPs’ concerns that these conversations might be intrusive or upset patients? |
| Summary | Key messages to share with HCPs to inform and guide their practice | Key messages to share with HCPs to inform and guide their practice |

Appendix 2: Topic guide for focus group with HCPs with experience of working in a UK NHS setting with adult patients who have an illness

| Professional experience of family-centred conversations | Current practice regarding identifying patients’ relationships with children and exploring what has been communicated to them about the adult’s illness  Experience during training or work in other contexts/settings |
| --- | --- |
| Perception of benefits and risks of family-centred conversation |  |
| Perceived obstacles to delivering family-centred communication in routine care |  |
| Format or content of training interventions | Experience and perception of successful training or implementation initiatives from other areas of practice development  Perception of what achieves a change in HCPs’ behaviour or practice |
| Support or resources required | What do HCPs need to address identified obstacles and facilitate family-centred communication |
